# Artificial Dentistry

**Published:** 1873-06

**Authors:** Frank A. Hunter


### ARTIFICIAL DENTISTRY.

BY FRANK A. HUNTER.

Should it be practiced as a speciality? What may be done to raise the standard of operations in this department?

On the subject now open for discussion I propose making only a few remarks in order to "start the ball rolling," then leaving to older and more experienced hands the pleasure of "keeping it going."

Artificial Dentistry—Artificial—contrived with skill or art. Dentistry—the art or practice of a dentist. Therefore Artificial Dentistry comprises *almost* the entire occupation of a dentist. I will however confine myself to the general acceptation of the term by the profession.

In the latter part of the 18th century the first porcelain teeth were made, but were not in general use for a considerable time thereafter; for in those days it was the custom to carve artificial teeth from bone, ivory, or the tooth of the hippopotamus, and in some cases setting human or sheep's teeth on a base of ivory.

Early in the 19th century, gold, silver, and platinum, were used as a base, with porcelain teeth rivited on, and later, moulded teeth with pins were introduced, and from that time improvement became the order of the day. Between 1845 and 1850 continuous gum work was introduced in an improved form, and was readily adopted by the advanced men of the profession ; and between that time and 1860 I venture to say, without fear of contradiction, that there was *more artistic* work made than ever before or since.

During that period you never heard the question asked, " Should it be practiced as a specialty ?" And why ? because men of acknowledged ability were not ashamed to do artificial work, as it requires as much, if not more skill and ingenuity, to construct an *artistic* set of teeth, than is generally employed in the operation of filling a tooth.

In 1858–59 rubber was introduced by the American Hard Rubber Co., as a base for artificial teeth, but its use did not become general until about 1860–61, then artistic dentistry received almost a fatal blow—then the gate was opened for men with a low order of talent to enter the profession, and by their competition endeavor to drive the accomplished dentist from one branch of his profession.

From its introduction to the present day it has been constantly on the increase, and now there are at least one hundred sets of teeth made on rubber to one on any other base. Why ? Because it is cheap. Why do the dentist's make it ? Because it is easy—because it requires little skill, and can be made for a small amount of money.

What artistic skill is displayed in the construction of a set of teeth on rubber, with moulded blocks ? Absolutely none.

A man who can not, or does not make artificial teeth on any other base than rubber is not worthy the name of dentist.

I do not take the extreme stand that some do, and say that rubber is not a fit thing for the mouth in any case, but I do say that there are very few cases where almost anything else would not be better—a partial set of teeth on rubber is execrable.

Should it be practiced as a specialty ? Certainly—rubber work.

What may be done to raise the standard of operations in this department ?

In the first place discard rubber and all bases on which we use block teeth.

Inform your patients, for in many cases it is for want of knowledge that they accept the worst for the best.

Let those that *used* to know how, return to thelr duty, and make for permanent cases gold or platina work—and let those that don't know how learn.

The cry of the majority of the people is stlll for rubber, but there is a minority that may be increased, who appreciate, and are willing to pay for first-class artificial dentistry if the facts are properly presented to them.

